# Transformation from acute promyelocytic leukemia to acute myeloid leukemia with a CEBPA double mutation: A case report and review of the literature

**DOI:** 10.1097/MD.0000000000024385

**Published:** 2021-02-05

**Authors:** Ye Sun, Chong Wang, Yongcheng Sun, Jiaping Wang, Chunmeng Rong, An Wu, Guifang Ouyang, Lixia Sheng

**Affiliations:** a Department of Hematology, Ningbo First Hospital; b Medical School of Ningbo University, Ningbo, Zhejiang Province; c Department of Hematology, the First Affiliated Hospital of Zhengzhou University, Zhengzhou, Henan Province, China.

**Keywords:** acute myeloid leukemia, acute promyelocytic leukemia, CCAAT/enhancer-binding protein alpha mutation, transformation

## Abstract

**Introduction:**

The transformation of acute promyelocytic leukemia (APL) to acute mononuclear leukemia during treatment is a rare clinical phenomenon, and no CCAAT/enhancer-binding protein alpha (CEBPA) double mutations have been reported.

**Patient concerns:**

A 42-year-old male was hospitalized for ecchymosis of the left lower limb for more than 1 month, gingival bleeding, and fatigue for 10 days, with aggravation of symptoms for 2 days.

**Diagnosis:**

A diagnosis of APL was based on bone marrow (BM) morphology, immunophenotyping, fusion gene analysis, and fluorescence in situ hybridization. At a 1-year follow-up of maintenance treatment, he developed thrombocytopenia and was diagnosed with acute myeloid leukemia (AML) with a CEBPA double mutation by BM morphology, immunotyping, chromosomal analysis, polymerase chain reaction, and next generation sequencing.

**Interventions:**

Complete remission of APL was achieved after all-trans retinoic acid and arsenic trioxide double induction therapy, followed by 2 cycles of mitoxantrone and cytarabine, and 1 cycle of idarubicin and cytarabine. Thereafter, sequential maintenance therapy of arsenic trioxide + all-trans retinoic acid + methotrexate was started. In the fourth cycle of maintenance therapy, APL was transformed into AML with a CEBPA double mutation. After 1 cycle of idarubicin and cytarabine, the patient achieved complete remission and received 3 cycles of idarubicin and cytarabine and three cycles of high-dose cytarabine as consolidation therapy.

**Outcomes:**

At present, the patient is in continuous remission with minimal residual disease negative for both of APL and AML.

**Conclusion:**

AML with a CEBPA double mutation after APL treatment is very rare, thus the prognosis of this event will require further observation.

## 1 Introduction

Acute promyelocytic leukemia (APL) is a common subtype of acute myeloid leukemia (AML) and was formerly one of the most fatal forms of AML. The introduction of all-trans retinoic acid (ATRA) in combination with arsenic trioxide (ATO) and other chemotherapies has resulted in complete remission rates of >90% and long-term remission rates of >80%.^[1]^ However, a very small number of leukemia subtypes can undergo disease transformation in response to chemotherapy or develop treatment-related secondary leukemia, which may be insensitive to chemotherapy, resulting in a poor prognosis. Hence, the clinical diagnosis and treatment of APL remain challenging. Here, we report a case of acute myelomonocytic leukemia with a double mutation to the CCAAT/enhancer-binding protein alpha (CEBPA) gene during maintenance therapy for APL and include a review of the current literature.

## 2 Case report

**Figure 1:**
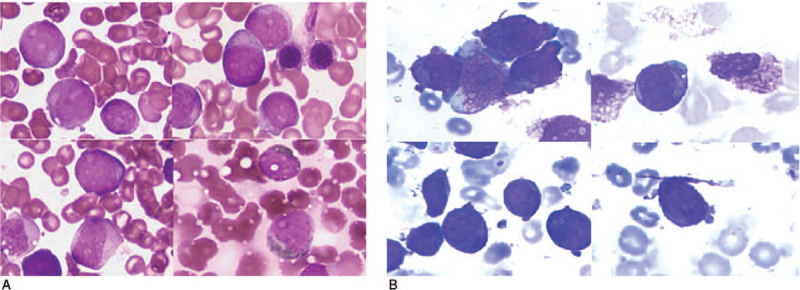
Bone marrow morphological characteristics of the patient at diagnosis of acute promyelocytic leukemia and conversion to acute myeloid leukemia. (A) Before transformation. (B) After transformation. (Gitter-Giemsa staining,× 1000).

**Figure 2:**
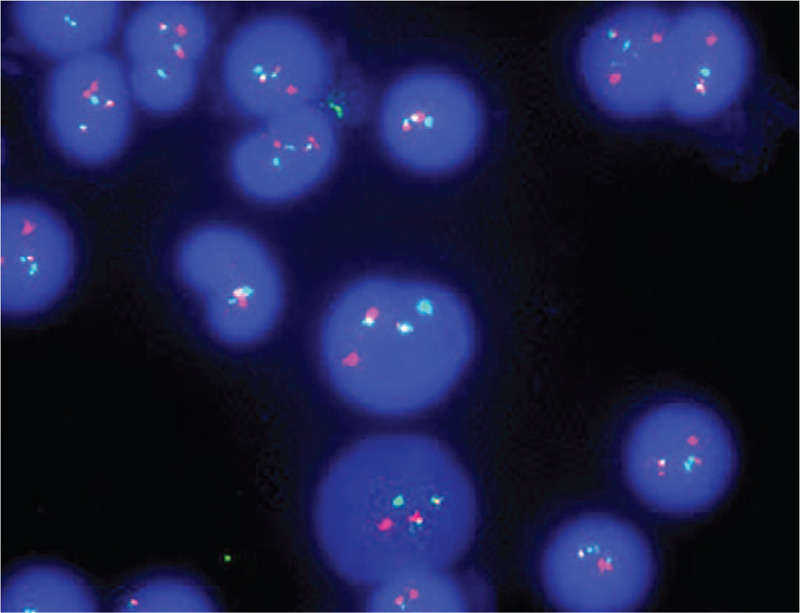
FISH detection of PML-RARα in the patient at diagnosis of acute promyelocytic leukemia. About 68% of 400 analyzed cells exhibited a red-green fusion signal representing the translocation of PML (green) and RARα (red) (DAPI staining,×1000).

The patient was a 42-year-old male with no previous exposure to industrial toxins or radioactive substances. In November 2018, he was hospitalized for ecchymosis of the left lower limb for more than 1 month, gingival bleeding, and fatigue for 10 days, with aggravation for 2 days. On physical examination, the patient appeared anemic with scattered spots of bleeding of the mucosa and the skin over the whole body, no abnormality in heart rhythm or lung auscultation, a soft abdomen, no palpation of the liver or spleen under the ribs, and no edema of either lower limb. The findings of initial blood chemistry analysis were as follows: white blood cell count, 8.1 × 109/L; hemoglobin concentration, 69 g/L; platelet count, 16 × 109/L; prothrombin time, 14.6 s, activated partial thromboplastin time, 21.4 s; thrombin time, 21.5 s; K+, 3.71 mmol/L; Na+, 141 mmol/L; Ca2+, 1.89 mmol/L; γ-glutamine acylase, 144 U/L; total protein, 57.2 g/L; and albumin, 33.2 g/L. Bone marrow (BM) analysis revealed abnormal promyelocytes (89.2%) of different sizes with quasi-round shapes and tumor-like processes on the edge of the cytoplasm, medium amounts of cytoplasm, blue staining, purplish red granules in the cytoplasm, round nuclei, visible depressions, loose and fine chromatin, and visible nucleoli. In addition, erythroid differentiation was inhibited, megakaryocytes were absent, and platelets were rare (Fig. 1A). Fluorescence in situ hybridization of the BM showed that 68% of the 400 cells analyzed within the detection range revealed the presence of the PML/RARa fusion gene (Fig. 2). Further genetic screening revealed mutations to NRAS (NM_002524,cxon2:c.G35A:p.G120rs121913237) and TET (NM_001127208,cxon11:c.A5284G:p.11762Y). Flow cytometry analysis of the BM revealed the presence of abnormal cells that were positive for CD117, CD13, CD33, CD38, CD64, CD123, CD58, and cMPO, with partial expression of CD71, but negative for CD19, CD3, and CD34. Based on these findings, the patient was diagnosed with APL (medium-risk group). After ATRA + ATO double induction therapy, the patient received 2 cycles of ATRA + mitoxantrone and cytarabine, and one cycle of ATRA + idarubicin and cytarabine. Afterward, the patient was negative for the PML-RARα fusion gene. Following remission, intrathecal chemotherapy was administered to prevent the onset of central nervous system leukemia. On April 2, 2019, the patient started an alternating maintenance treatment regimen of ATO + ATRA + methotrexate. Before the fourth cycle of maintenance therapy on January 27, 2020, a BM examination and fusion gene analysis revealed complete remission of APL. On April 14, 2020, during the methotrexate regimen, the results of a routine blood test were as follows: white blood cell count, 4.01 × 109/L; percentage of monocytes, 15%; hemoglobin concentration, 56 g/L; platelet count 76 × 109/L; prothrombin time, 11.0 s; activated partial thromboplastin time, 31.5 s; thrombin time, 16.1 s; K+, 4.35 mmol/L; Ca2+, 2.31 mmol/L; alanine aminotransferase, 12 U/L; aspartate transaminase, 14 U/L; creatinine, 66 μmol/L, total protein, 57.2 g/L; and albumin, 45.3 g/L. A physical examination revealed no anemia, no sites of bleeding on the skin or mucosa, no abnormality in heart rhythm or lung auscultation, a soft abdomen, liver and spleen not palpable under the ribs, and no edema of either lower limb. The patient had no symptoms of altered consciousness at that time, so a bone puncture was performed immediately. BM morphological analysis revealed that 31.0% of the monocytes were primitive and immature with varied cell sizes, medium to rich cytoplasm, large nuclei, fine chromatin that was twisted and folded, visible nucleoli, granulocyte content of 6.5%, some vacuoles throughout the cytoplasm, active red proliferation, visible plasma, reticular cells with normal morphologies, 237 megakaryocytes inone smear, and visible platelets (Fig. 1B). Flow cytometry revealed that 44.75% of the primitive immature cells were nucleated and positive for CD34, CD38, CD13, CD15, CD117, CD33, CD7, and HLA-DR, but negative for CD3 and CD19. Myeloid expression was abnormal, which conformed to the AML phenotype. The PML-RARα gene was negative by fluorescence in situ hybridization analysis. Qualitative detection of 38 fusion genes specific to leukemia was also negative. Gene mutation analysis by next generation sequencing identified an insertion mutation to the CEBPA gene (c.912_913insTTG (p.Lys304_Gln305insLeu; heterozygous mutation frequency, 28.5%) and a code shift mutation (c.326del (p.Pro109ArgfsTer51; heterozygous). BM cytogenetic studies revealed a normal karyotype of 46, xy (20). The combined results of the physical examination and laboratory results suggested a diagnosis of AML and acute myelomonocytic leukemia with a CEBPA double mutation (low risk group). Therefore, induction chemotherapy was started with a regimen of idarubicin and cytarabine (idarubicin at 10 mg/m2 on days 1–3; and cytarabine at 100 mg every 12 hour on days 1–7). Reexamination of the BM at 14 days after chemotherapy showed that the patient had achieved complete remission. Thereafter, the patient received 3 cycles of idarubicin and cytarabine, and 3 cycles of high-dose cytarabine as consolidation therapy. At present, the patient is in continuous remission with minimal residual disease for both APL and AML.

## 3 Discussion

It is rare for leukemia to undergo lineage transformation during clinical treatment and there is no clear evidence to confirm the underlying mechanism. Moreover, the development of secondary myeloid tumors after APL therapy is also very rare. For example, Montesinos et al^[2]^ reported 17 cases of secondary myeloid tumors, including AML and myelodysplastic syndrome, among 918 APL patients after treatment. The cumulative 6-year incidence of secondary myeloid tumors was 2.2%, with a higher incidence in APL patients aged >35 years, while in the low risk group, common cytogenetic abnormalities included –5, –7, and 11q23 rearrangements. The prognosis of secondary treatment-related myeloid tumors in APL is often poor, with a median survival time of only 10 months. Although the pathogenesis of secondary treatment-related leukemia remains unclear, 3 main hypotheses have been proposed:

(i) the use of cytotoxic drugs, especially topoisomerase inhibitors, alkalinizing agents, and anthracyclines,^[3,4]^ leading to changes in DNA structure and secondary changes, most of which are treatment-related myelodysplastic syndrome or AML;^[5,6]^
(ii) APL accompanied by other clones in the early stage of the disease, but masked by the dominant clones, as the application of ATRA, ATO, and chemotherapy eliminate abnormal promyelocytes, so that other clones have a better chance of survival; and
(iii) the inherent lineage plasticity of primitive leukemia cells determines the potential for reprogramming under lineage-specific pressure^[7,8]^ and more frequent lineage switching with specific genetic subtypes of leukemia, such as mixed lineage leukemia rearrangement, which may have greater inherent plasticity.^[9]^ In the present case, the onset of secondary myeloid leukemia was earlier, as the switch to AML occurred only 17 months after complete remission of APL, which is a much shorter period than the median time of 44 months reported in the literature.^[2]^ A retrospective study conducted by Wang et al^[6]^ reported treatment-related myeloid tumors in 67 cases that most commonly occurred at about 3 years after complete remission of APL. In view of the short period of remission during treatment in this case, it was difficult to differentiate treatment-related secondary myeloid tumor formation from APL recurrence with a series of transitions. Moreover, a retrospective analysis of 651 AML patients conducted by Wang et al^[10]^ found fewer favorable genetic molecular events in elderly patients with AML and secondary AML patients, such as a CEBPA double mutation, runx1-runxt1t1, etc. A double mutation to the CEBPA gene is detected in leukemia cells during AML transformation, and some cases before transformation to APL were accompanied with a double mutation to the CEBPA gene.^[11]^ However, our patient was negative for the CEBPA double mutation at the first onset, which does not support the possibility of evolution from APL to AML. The CEBPA double mutation is often associated with a good prognosis of AML. Patients with a CEBPA double mutation are classified as low risk. However, in this patient, AML was secondary to APL. As reported in the literature, the prognosis of APL secondary to treated-acute myeloid leukemia is poor and survival is relatively short. Although the patient achieved complete remission after 1 course of chemotherapy, his long-term survival remains to be further investigated, as it is unclear whether the choice of follow-up consolidation therapy should be medium dose cytarabine-based chemotherapy or allogeneic hematopoietic stem cell transplantation.

The formation of a secondary myeloid tumor after APL treatment is a rare clinical phenomenon, which usually occurs at about 3 years after disease remission, although early development of a secondary myeloid tumor is not impossible. Also, it remains uncertain whether the prognosis of secondary AML with a CEBPA double mutation after APL is equivalent to that of newly diagnosed AML with a CEBPA double mutation, thus additional clinical cases are needed. The mechanism underlying secondary AML after APL therapy is unknown, but it is believed that with the development of genetic cytology and the accumulation of clinical cases, more accurate mechanisms will be identified and individualized prevention and treatment regimens will be clinically available.

## Author contributions

**Conceptualization:** Guifang Ouyang.

**Data curation:** Ye Sun, Chong Wang, Yongcheng Sun, Jiaping Wang, Lixia Sheng.

**Formal analysis:** Chunmeng Rong, An Wu.

**Funding acquisition:** Lixia Sheng.

**Methodology:** Lixia Sheng.

**Writing – original draft:** Ye Sun.

**Writing – review & editing:** Lixia Sheng.
